# Cameras or *Camus*? Comparing Snow Track Surveys and Camera Traps to Estimate Densities of Unmarked Wildlife Populations

**DOI:** 10.1002/ece3.70747

**Published:** 2024-12-23

**Authors:** Scott J. Waller, Mark Hebblewhite, Jedediah F. Brodie, Svetlana V. Soutyrina, Dale G. Miquelle

**Affiliations:** ^1^ Wildlife Conservation Society New York New York USA; ^2^ Wildlife Biology Program University of Montana Missoula Montana USA; ^3^ Division of Biological Sciences University of Montana Missoula Montana USA; ^4^ Institute for Biodiversity and Environmental Conservation Universiti Malaysia Sarawak Kota Samarahan Sarawak Malaysia; ^5^ Sikhote‐Alin Biosphere Zapovednik Primorsky Krai Russia

**Keywords:** Amur tiger, camera traps, Formozov‐Malyushev‐Pereleshin (FMP) method, prey densities, snow track surveys, unmarked populations

## Abstract

Population density is a valuable metric used to manage wildlife populations. In the Russian Far East, managers use the Formozov‐ Malyushev‐Pereleshin (FMP) snow tracking method to estimate densities of ungulates for hunting management. The FMP also informs Amur tiger (*Panthera tigris altaica*) conservation since estimates of prey density and biomass help inform conservation interventions. Yet, climate change and challenges with survey design call into question the reliability of the FMP. Camera traps offer a promising alternative, but they remain unexplored for monitoring tiger prey density. Over three years (2020‐2022), we used the FMP and camera‐based methods to estimate densities of four prey species of the Amur tiger in the Sikhote‐ Alin Biosphere Reserve, Russian Far East: wild boar (*Sus scrofa*), red deer (*Cervus canadensis*), roe deer (*Capreolus pygargus*), and sika deer (*Cervus nippon*). We compared FMP results from snow track survey routes either along trails, or along routes representative of the study area, and estimates derived from camera data using the random encounter model (REM), space‐to‐event model (STE), and time‐to‐event model (TTE). We found that density estimates from representative routes were typically lower than routes along trails and indicated different relative densities of prey. Density estimates from camera traps and representative track surveys were generally similar with no significant relative bias, but precision was poor for all methods. Differences between estimates were amplified when converted to prey biomass, particularly with larger, more abundant prey, which poses a challenge for their utility for tiger managers. We conclude camera traps can offer an alternative to snow track surveys when monitoring unmarked prey, but we caution that they require considerably more resources to implement. Tiger managers should be especially cautious when extrapolating density to estimates of prey biomass, and we encourage future research to develop more robust methods for doing so.

## Graphical abstract

Waller et al. used camera traps to estimate the densities of four ungulate species in the Russian Far East and compared these estimates with those from the traditional Formozov snow tracking approach. The authors found that both approaches can provide consistent estimates of population density, but the time and resource demands of snow tracking were considerably less. They also show that more rigorous methods need to be developed to convert estimates of density to estimates of prey biomass, which is a valuable metric used in carnivore conservation.

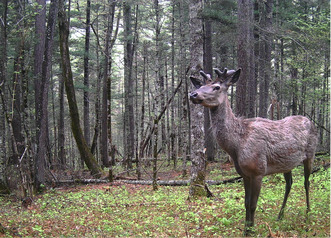

## Abstract (ru)

Плотность популяции ‐ важный показатель, используемый для управления популяциями диких животных. На Дальнем Востоке России в целях управления охотничьим хозяйством для оценки плотности популяций копытных животных специалисты используют метод зимних следовых учетов (ЗМУ), основанный на формуле Формозова‐Малышева‐Перелешина (ФМП). Метод ЗМУ также используется для сохранения амурского тигра (*Panthera tigris altaica*), поскольку оценки плотности и биомассы его видов‐жертв помогают обосновать проведение природоохранных мероприятий. Тем не менее, изменение климата и проблемы с организацией исследований ставят под сомнение надежность ЗМУ. Фотоловушки являются многообещающей альтернативой, но они пока остаются неизученными инструментом для мониторинга плотности популяций видов‐жертв тигра. В течение трех лет (2020‐2022 гг.) мы использовали ЗМУ и фотоловушки для оценки плотности популяций четырех видов‐жертв амурского тигра ‐ кабана (Sus scrofa), изюбря (Cervus canadensis), косули (Capreolus pygargus) и пятнистого оленя (Cervus nippon) в Сихотэ‐Алинском биосферном заповеднике на Дальнем Востоке России. Мы сравнили результаты ЗМУ, полученные на маршрутах, проложенных вдоль троп, или на маршрутах, репрезентативных для территории исследования, и оценки, полученные по данным с фотоловушек с использованием модели случайных встреч (REM), модели «пространство‐событие» (STE) и модели «время‐событие» (TTE). Мы обнаружили, что оценки плотности, полученные на репрезентативных маршрутах, обычно ниже, чем на маршрутах, проложенных вдоль троп, и указывают на разную относительную плотность видов‐жертв. Оценки плотности, полученные с помощью фотоловушек и на маршрутах, репрезентативных для территории исследования, были в целом схожими, без значительных относительных отклонений, но точность этих оценок была низкой для всех методов. Различия между оценками усиливались при пересчете на биомассу видов‐жертв, особенно если речь шла о более крупных и многочисленных животных, что ставит под сомнение полезность таких данных для специалистов по сохранению тигра. Мы пришли к выводу, что фотоловушки могут стать альтернативой следовым учетам при мониторинге животных, особей которых невозможно идентифицировать индивидуально, но предупреждаем, что для их применения требуется значительно больше ресурсов. Специалистам по сохранению тигра следует быть особенно осторожными пересчете показателей плотности на биомассу видов‐жертв, и мы призываем к проведению дальнейших исследований для разработки более надежных методов для этого.

## 1 Introduction

Estimates of population density are invaluable to wildlife managers seeking to make evidence‐based decisions about ungulate management. Many ungulates provide benefits to people (Nasi, Taber, and Van Vliet 2011, Pascual‐Rico et al. 2021), and population density and related metrics (such as biomass density) are important indicators that help identify thresholds of human–wildlife coexistence (Garshelis, Noyce, and St‐Louis 2020; Carpio, Apollonio, and Acevedo 2021) and maximum sustainable yields to meet both human well‐being and ungulate conservation goals (Robinson and Bennett 2004; Wilkie, Wieland, and Poulsen 2019). Beyond their importance to humans, many ungulates play important roles in their ecosystems, yet face pressing anthropogenic threats (Nasi, Taber, and Van Vliet 2011; Brodie et al. 2015; Ripple et al. 2015). Estimates of population density can help identify trends and the outcomes of conservation actions to recover these ungulates (Saisamorn et al. 2024). Finally, many ungulates are important prey species for large carnivores and therefore are a key part of conservation strategies to recover those carnivores (e.g., WWF et al. 2018; Tiger Conservation Coalition 2021).

As an example, density estimates of preferred prey are often valuable to tiger conservationists and can help inform the recovery of wild tigers across their range (Walston et al. 2010; Gray et al. 2023; Sanderson et al. 2023). When reintroducing tigers to a new landscape, prey densities can help identify a suitable site for release (Miquelle et al. 2024). In other instances, concomitant estimates of tiger density and prey biomass—extrapolated from prey densities—can determine whether prey availability is limiting tiger recovery (e.g., Upadhyay et al. 2019; Qi et al. 2021; Saisamorn et al. 2024).

Biomass of preferred prey is usually estimated by multiplying estimates of prey population density by literature‐derived average weights of each prey species (e.g., Miquelle et al. 2010; Zhang, Zhang, and Stott 2013; Simcharoen et al. 2014) or, when data allow, based on direct observations adjusted according to sex and age class proportions (Schaller 1967; Karanth and Sunquist 1992). But to the frustration of conservationists, estimating prey densities of tigers has often proven challenging. Individuals of preferred prey, such as wild boar ( *Sus scrofa* ) and sambar deer ( *Rusa unicolor* ) (Hayward, Jedrzejewski, and Jedrzewska 2012), typically cannot be uniquely identified, precluding the application of capture–recapture models as used to monitor densities of tigers (Karanth 1995; Efford, Borchers, and Byrom 2009; Royle et al. 2009). Line‐transect distance sampling is limited to only a few places where animals are easily visible, prey densities are high, and animals tend not to flee from humans (Karanth and Nichols 2002; Harihar, Pandav, and Macmillan 2014; Karanth, Kumar, and Karanth 2020). Most ecosystems do not share these attributes, and managers must use other methods.

In snow‐covered regions of the world, such as Finland and Russia, wildlife managers use snow tracking techniques based on the Formozov‐Malyushev‐Pereleshin (FMP) method (Formozov 1932; Stephens, Zaumyslova, Hayward et al. 2006) to estimate prey densities for hunting management (Helle, Ikonen, and Kantola 2016; Razenkova et al. 2023). In the Russian Far East, wildlife managers also use the FMP to improve conservation of the Amur tiger (*P.t. altaica*). The FMP has provided valuable long‐term datasets in Russia (e.g., Stephens, Zaumyslova, Hayward et al. 2006) and can be both cheaper and result in more detections of study species than other methods like aerial surveys (Keeping et al. 2018; Ahlswede et al. 2019). But the approach is prone to potential bias caused by convenience sampling (Stephens, Zaumyslova, Hayward et al. 2006): many surveys are only conducted along roads and trails as random sampling off‐trail is considerably more challenging. This violates the critical assumption of representatively (e.g., randomly) sampling the variety of landscape features in a study area that affect the local abundance and movement of the study species. Moreover, snow track surveys depend critically on consistent, recurring snowfall. This has become a major concern in northern temperate regions like the Russian Far East, with climate change leading to warming temperatures and changing precipitation (Stephens, Zaumyslova, Hayward et al. 2006; IPCC 2022). The issues with convenience sampling and snow conditions call into question the reliability of the FMP method in the Russian Far East in the future. There is a need to both evaluate the potential bias caused by conducting track surveys only along linear features, as well as to find alternative, climate‐independent methods that can be used to estimate densities of prey.

Camera traps (“cameras” hereafter) offer a promising alternative to snow tracking as many managers are already familiar with camera trap technology and logistics. Several statistical models have been proposed in the last 15 years to estimate densities of unmarked populations by extrapolating density within the collective sampled areas in front of cameras (“viewsheds”) (reviewed by Gilbert et al. 2020). The most commonly used methods include the Random Encounter Model (REM) (Rowcliffe et al. 2008), the Random Encounter and Staying Time model (REST) (Nakashima, Fukasawa, and Samejima 2018), Camera Trap Distance Sampling (CT‐DS) (Howe et al. 2017), and the Space‐To‐Event and Time‐To‐Event models (STE and TTE) (Moeller, Lukacs, and Horne 2018). These models (collectively, “viewshed density estimators,” Moeller et al. 2023) have been used to estimate densities of both carnivore (Cusack et al. 2015; Loonam, Ausband, et al. 2021) and herbivore species (Morelle et al. 2020; Palencia et al. 2021; Lyet et al. 2023) across diverse ecosystems, and the number of tests and validations of these models is growing every year. Despite their increasing use, all models assume that cameras representatively sample the landscape (i.e., are not placed on linear features), and they require great effort to achieve acceptable levels of precision for low‐ and medium‐density populations (Cappelle et al. 2021; Morin et al. 2022). These challenges together may limit their feasibility for monitoring densities of tiger prey, which often occupy rugged terrain at relatively low densities.

In this study, we evaluated the use of snow track surveys and cameras to estimate population densities of the four main prey species of the Amur tiger (Miquelle et al. 1996, 2010; Kerley et al. 2015): wild boar, red deer ( *Cervus canadensis* ssp. *xanthopygus*), roe deer ( *Capreolus pygargus* ), and sika deer ( *Cervus nippon* ). Over three study years (2020–2022), we conducted winter track surveys using both conventional (i.e., along roads and trails) and representative (i.e., random) survey designs to assess the potential bias caused by conventional surveying. We also deployed cameras using a systematic random sampling design, then compared FMP estimates to those from the REM, STE, and TTE. Finally, we compared estimates of prey biomass when converted from prey density estimates from different methods. Together, these efforts provide a comprehensive assessment of the value and applicability of both FMP and camera‐based methods to estimate prey densities for tiger conservation in the Russian Far East.

## 2 Methods

### 2.1 Study Area

**FIGURE 1:**
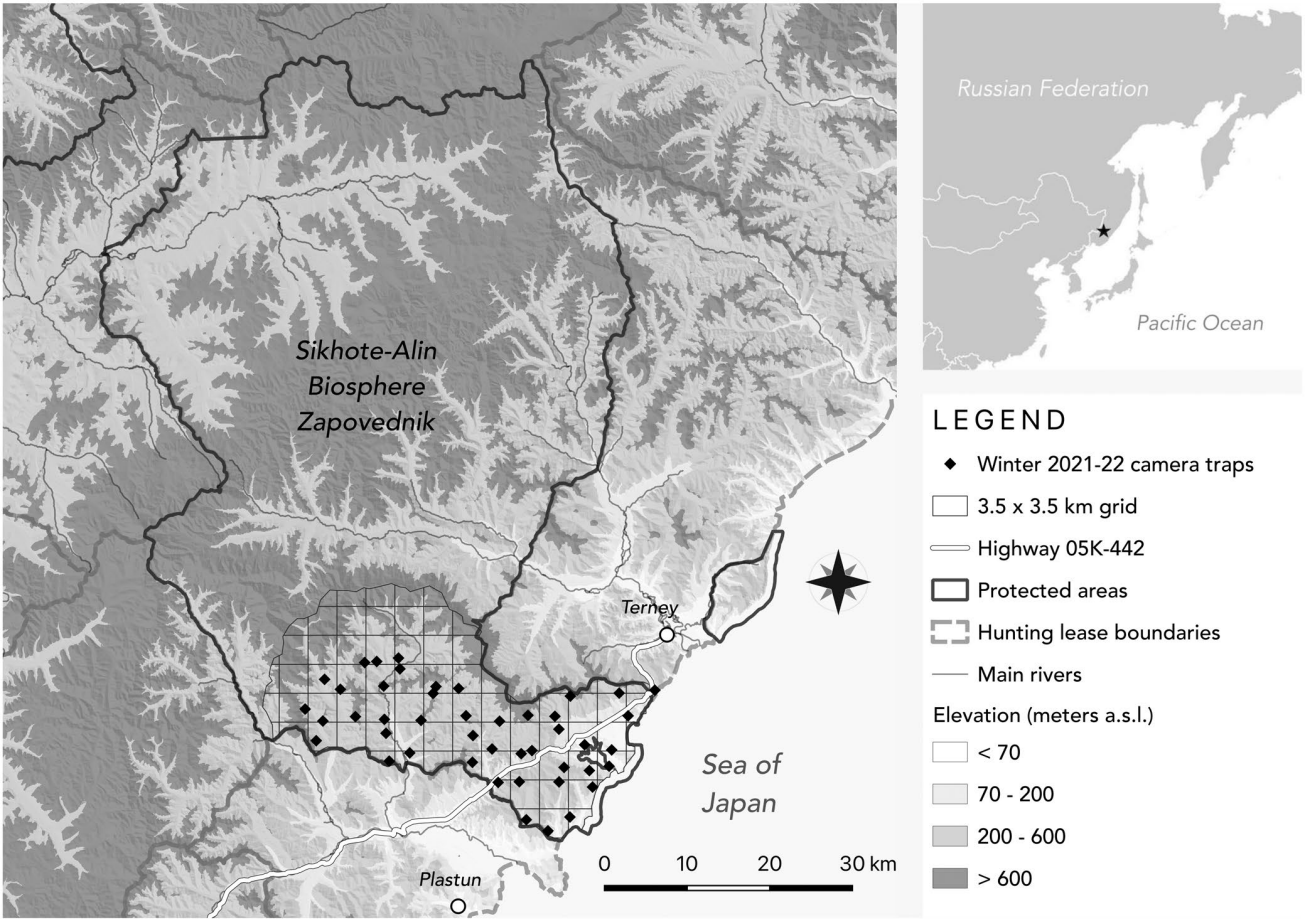
Map of the Sikhote‐Alin Biosphere Zapovednik and adjacent Terney Hunting Lease in central Sikhote‐Alin, Russian Far East. Snow track surveys were conducted and random cameras deployed in the 3.5 × 3.5 km grid in the southern portion of SABZ. Elevation bins were chosen to illustrate main ridges of the Sikhote‐Alin mountains. Cameras deployed during winter 2021–2022 are shown as example locations.

We conducted this study in the 4016 km^2^ Sikhote‐Alin Biosphere Zapovednik (hereafter “Zapovednik”), Primorsky Krai, Russian Far East (Figure 1). The Zapovednik is named after the Sikhote‐Alin Mountains, a low‐elevation range running northeast along the Sea of Japan and through the reserve. Summers in the Zapovednik are hot and wet while winters are relatively cold and dry (Miquelle et al. 2010). The reserve is mostly forested, with coastal Mongolian oak ( *Quercus mongolica* ) and mixed hardwood forests shifting to forests of Korean pine ( *Pinus koraiensis* ) and deciduous species further inland. These forest transitions result in important spatial variation in mast crop availability for wildlife, as acorns and pine nuts are dominant sources of food for much of the wildlife community (Heptner, Nasimovich, and Bannikov 1988; Waller et al. 2024). Our study to estimate prey densities took place in a roughly 500 km^2^ area within the southern portion of the Zapovednik. We chose this study area because it was more accessible relative to other parts of the reserve, and covered a diverse range of forest types common in the coastal Sikhote‐Alin where the FMP is most challenged by inconsistent snowfall and camera traps therefore might be the most useful for prey surveys.

### 2.2 Camera Trap Study Design and Effective Detection Distance

We deployed cameras using a systematic random sampling design over 3 years: spring 2020, winter 2020–2021, and winter 2021–2022 (Figure 1). Each year, we randomly deployed one camera in each cell of a rectangular 3.5 × 3.5 km grid drawn over our study area; this cell size is roughly the size of a female red deer's home range (Dou et al. 2019). We randomly generated one camera location in each cell, then excluded cameras that were not within the bounds of our study area. In the field, cameras were deployed as close to the randomly generated coordinate as possible (adjustments were sometimes necessary to find a tree of sufficient size for strapping the camera or to avoid steep cliffs). We used a variety of camera brands and models in winter 2019–2020, but only two models during winters 2020–2021 and 2021–2022 (Appendix A). Cameras were placed 1 m above the ground, facing north to minimize glare from the sun, and were active 24 h per day. Camera settings were configured to take rapid bursts of three photos at each capture with no delay. No baits or lures were used.

All viewshed density estimators require measurements of the area effectively sampled by cameras to extrapolate detections within the viewshed across the study area. With motion‐trigger photography, the area effectively sampled by cameras is a complex parameter that depends on multiple factors such as animal speed and size, ambient temperature, sensor quality, and understory vegetation characteristics (Hofmeester et al. 2019; Moeller et al. 2023). The most important factor is the distance of animals to the camera: animals that are further away have a decreased capture probability. To account for this, Rowcliffe et al. (2011) developed the effective detection distance (EDD), which uses estimated distances to animals from the camera to account for imperfect detection. We applied a combination of automated and manual distance sampling techniques introduced by Haucke et al. (2021) to estimate the distances to detected animals during our last field season. These were then used to estimate EDD for each species using the methods described in Rowcliffe et al. (2011) and Howe et al. (2017). Then, we used simple sector geometry to calculate the effective detection area in each year based on the estimated EDD and the average viewable angle across camera models for each year (Moeller et al. 2023). Additional details about our methodology are provided in Appendix B.

### 2.3 Formozov‐Malyushev‐Pereleshin Model

The FMP method estimates density by relating the encounter rate of fresh (< 24 h old) tracks observed along walked/skied transects with independent estimates of the study species' daily travel distance. In the FMP formula, the density *D* of a population is estimated by the following:

(1) $$D=\frac{\pi }{2}\;\frac{x}{S\;\hat{M}}$$

where *x* is the total number of tracks encountered, *S* is the total length (km) of all transects, and $\hat{M}$ is the study species' daily travel distance (km). The term $\frac{\pi }{2}$ relates the species' daily travel distance to the probability of encountering a track along the surveyed transects, integrated over all possible angles of intersection between animal's movement paths and the transects (Stephens, Zaumyslova, Miquelle et al. 2006). The FMP method assumes the following: (1) geographic and demographic closure; (2) the species and age of tracks are identified without error; (3) animals move independently of transects; and (4) transects are representative of the study area.

Historically, the Zapovednik has conducted surveys only along roads and trails due the difficulties of conducting surveys off‐trail. However, this surely violates Assumptions (3) and (4) above (Stephens, Zaumyslova, Miquelle et al. 2006). We therefore estimated density of prey species first with the conventional survey routes along roads and trails (“conventional surveys”), and second, with survey routes representative of the study area and independent of animal movement (“random surveys”). These random survey routes were not generated in a truly random fashion, but were hand‐drawn with input from Zapovednik staff to be distributed across the entire study area, reasonably accessible, and representative of the slopes, elevations, aspects, and cover types of the study area, all of which have been shown to influence the local abundance and distribution of prey (Hebblewhite et al. 2014). A map of both conventional and random survey routes in 2021–2022, along with random camera placements in the same year, is provided in Appendix C. For each survey, teams of two surveyors walked, skied, or snowmobiled transects and recorded the number of fresh tracks of our study species. All tracks encountered along the transect were counted, including re‐crossings of the same individual (Keeping and Pelletier 2014). To inform the daily travel distance parameter, we used estimates and associated error reported in Stephens, Zaumyslova, Hayward et al. (2006) based on decades of tracking by SABZ staff of individual animals' movement paths through the snow. We used nonparametric bootstrapping (Efron and Tibshirani 1993) to estimate 95% confidence intervals of each density estimate. First, encounter rates ($\frac{x_{j}}{S_{j}\;}$) of each track *j* were sampled with replacement along with a daily travel distance ($\hat{M}$) drawn from the sample error reported by Stephens, Zaumyslova, Hayward et al. (2006). These parameter values were used for 1000 estimates of density from which we calculated the mean, 95% confidence intervals, and relative standard error (RSE).

### 2.4 Random Encounter Model

The REM was derived from both ideal gas theory (Hutchinson and Waser 2007) to describe animal movements and from the FMP formula (Stephens, Zaumyslova, Miquelle et al. 2006) to relate animal movement to the probability of intersection with a transect. Rowcliffe et al. (2008) proposed the following equation to estimate density *D*:

(2) $$D=\frac{y}{t}\;\frac{\pi }{\mathrm{vr}\left(2+\theta \right)}$$

where *y* is the number of independent detections of the individuals, *v* is the average daily travel distance (km) of the animal, *t* is the total number of days that cameras were deployed, and *r* and θ are the average radius (km) and angle of the registration zone in front of the cameras (Moeller et al. 2023). The assumptions of the REM are similar to those of the FMP: (1) geographic and demographic closure; (2) the ideal gas model sufficiently describes the movement of animals (which results in them being Poisson distributed; Hutchinson and Waser 2007); (3) detections of animals are independent; (4) animals move independently of camera traps; and (5) the site camera samples are representative of the study area. We considered detections of individuals of the same species 30 min apart to be independent. Estimates of daily travel distance were derived as for the FMP. Note that because we used snow track‐based estimates of distance traveled, we did not have to adjust detections by the study species' activity levels (Rowcliffe et al. 2014). We used 1000 iterations of nonparametric bootstrapping of both the encounter rate $\frac{y_{i}}{t_{i}}$ of each camera *i* and the daily travel distance *v* to generate a bootstrapped distribution of density estimates. This distribution gave us point estimates of density, 95% confidence intervals, and RSE.

### 2.5 Bootstrap Application of the Space‐To‐Event Model

As presented in Moeller, Lukacs, and Horne (2018), if animals are assumed to be Poisson‐distributed across the study area, then the amount of sampled area (*S*) until a detection is obtained is exponentially distributed:

(3) $$S\sim \mathrm{Exp}\left(\lambda \right)$$

where the rate parameter, lambda $\left(\lambda \right)$, is the average number of animals per unit area (km^2^). Cameras are randomly selected at regular intervals of time (“occasions”), and their viewshed areas are summed until a camera is selected that has an animal detection. With many occasions, these observed “space‐to‐events” form an exponential distribution from which lambda and associated variance are estimated by maximum likelihood estimation. If there are still no animal detections after randomly sampling all cameras during an occasion, then that occasion is right‐censored. So long as these occasions are “instantaneous” in time (i.e., at the finest temporal resolution of sampling, typically 1 s), the STE model estimates density without requiring independent estimates of animal movement rate. The STE model assumes the following: (1) geographic and demographic closure; (2) detections of animals at occasions are independent; (3) instantaneous sampling of occasions; (4) animals are Poisson‐distributed; (5) animals move independently of camera traps; and (6) the sites cameras sample are representative of the study area. The STE also assumes (7) perfect capture probability of animals in front of the camera.

The STE was initially designed using time‐lapse photography to best meet the assumption of perfect capture probability (Moeller, Lukacs, and Horne 2018). However, because of the expected low densities of prey, our camera data were collected using motion‐trigger photography, which violates this assumption. As such, we used a motion‐trigger application of the STE proposed by Lyet et al. (2023) that addresses the difficulties of applying the time‐lapse‐based STE to motion‐trigger data. This development of the STE uses a bootstrap approach to estimate density many times, with occasions starting at different times for each iteration. A final, mean estimate and 95% confidence intervals are then taken from the resulting bootstrapped distribution. We used 3‐min occasions for most density estimates, but if the proportion of estimates without any detections was greater than 0.05, we reduced the occasion length (e.g., to 1 min) until the proportion was 0.05 or less (Lyet et al. 2023). Because the STE does not account for animal movement and because we used motion‐trigger photography, we adjusted STE estimates by the inverse of activity levels, following methods of Rowcliffe et al. (2014). To meet the assumption of instantaneous sampling, we used a 1‐s sampling window at each occasion.

### 2.6 Time‐To‐Event Model

Instead of instantaneously sampling across all cameras on each occasion like the STE, the TTE samples space at each individual camera during shorter, regular intervals of time (“periods”) within each occasion. If the length of these periods is only long enough for animals to cross the viewshed of the camera, then the number of periods (i.e., the amount of sampled space at one camera) until a detection will be exponentially distributed (Moeller, Lukacs, and Horne 2018):

(4) $$T\sim \mathrm{Exp}\left(\lambda \right)$$

Lambda, the average number of animals in a camera's sampled area, is estimated with the exponential likelihood from the observed times‐to‐event of the species of interest at each camera. The TTE assumes the following: (1) geographic and demographic closure; (2) detections of animals in each occasion are independent; (3) animals are Poisson‐distributed across the cameras' viewsheds; (4) animals move independently of camera traps; and (5) cameras are representative of the study area. The TTE also requires estimates of animal movement rate to determine the period length. As with the FMP and REM, we used estimates of daily travel distance based on snow tracking and the widest part of the estimated effective detection area to determine the period length. Past research has used maximum likelihood estimation to estimate sampling variance following Moeller, Lukacs, and Horne (2018). However, to be consistent with other estimators, we used bootstrap resampling of cameras to develop a distribution of 1000 TTE estimates from which we calculated the mean density, 95% confidence intervals, and RSE.

### 2.7 Comparing Estimates of Prey Density and Biomass

In most previous studies, density estimates were compared by visually evaluating the overlap of 95% confidence intervals (e.g., Loonam, Ausband, et al. 2021; Lyet et al. 2023) or rarely through statistical tests (Palencia et al. 2021). In our case, we were specifically interested in how several camera‐based methods compared to FMP estimates from representative surveys. We therefore regressed camera‐based density estimates against FMP density estimates, such that 100% alignment between the methods would result in an intercept of 0 and a slope of 1 (i.e., no relative bias relative to the FMP estimate, and consistent similarity across the range of values). Following previous studies of Amur tigers (Miquelle et al. 2010; Zhang, Zhang, and Stott 2013), we then converted estimates of prey density to prey biomass (kg/km^2^) by multiplying point estimates of density from different methods by the average female body weights of prey species from the literature (Bromley and Kucherenko 1983). Female body weights were chosen as representing the average weight for the species, given the larger size of males and smaller size of young and subadults. As explained in other studies (e.g., Karanth and Sunquist 1992), we used point estimates as representations of the most likely prey density because the amount of error in our density estimates would have rendered all comparisons insignificant.

### 2.8 Data Processing and Analysis

All analyses were conducted using the R programming language (R Core Team 2023). We sorted camera trap images by species and removed images in which animals were obviously reacting to camera traps, as these detections violate the assumption that animals move independently of camera traps. We converted these images to detection data in R using the *camtrapR* package (Niedballa et al. 2016). For our study period, we chose a roughly 60‐day window when detections of each prey species were most consistent over time, and thus most likely to meet model assumptions about animal movement and detection. These periods were also either close in time or directly overlapped the snow track surveys. To estimate densities, we developed our own code for REM and FMP density estimates. For STE estimates, we adopted code provided by Lyet et al. (2023). We estimated activity levels using the *Activity* package (Rowcliffe et al. 2014). For TTE estimates, we used the *spaceNtime* package (Moeller and Lukacs 2021), then wrote our own code to produce bootstrapped density distributions.

## 3 Results

### 3.1 Survey Effort

We conducted 103 km of conventional winter track surveys in winter 2019–2020, 117 km during the winter 2020–2021, and 112 km in winter 2021–2022. We conducted 64 km of random track surveys in February–March 2020 and 166 km in February 2022. Because of the COVID‐19 pandemic, we were unable to conduct random surveys in winter 2020–2021. After removing malfunctioning cameras from our camera surveys, we obtained data from 31 cameras in the first year (1375 trap nights across 54 days, February 01, 2020 to March 26, 2020), 36 cameras in the second year (2223 trap nights across 62 days: December 10, 2020 to February 10, 2021), and 41 cameras in the third year (2583 trap nights across 62 days; December 01, 2021 to February 01, 2022). We note that in the second and third years, part of this increase in survey effort was thanks to better coverage of the western part of our study area, also the most difficult area to access.

### 3.2 Estimates of Prey Density and Biomass

**FIGURE 2:**
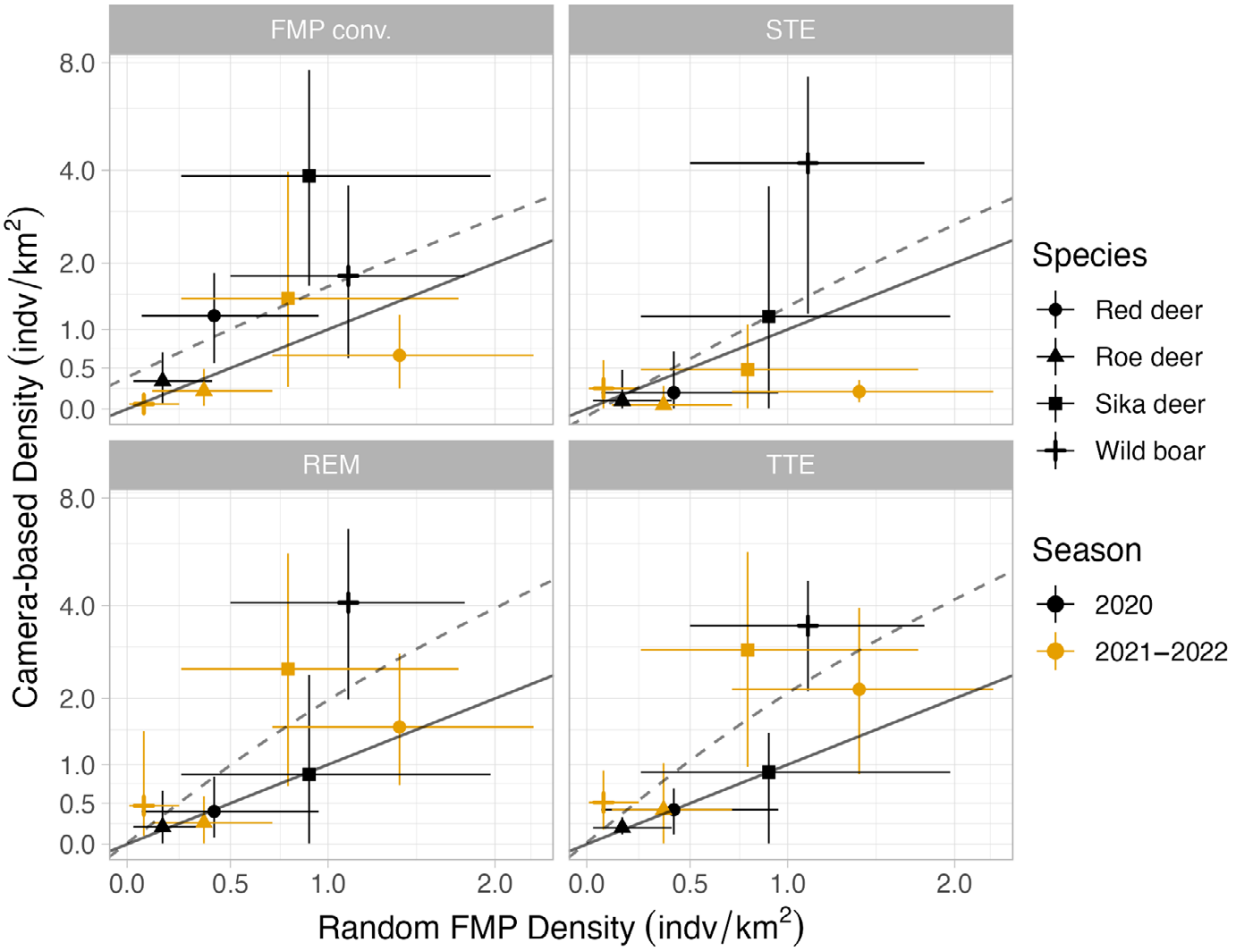
Relationship between estimates of population density from random FMP track surveys (*x*‐axis) and other methods (*y*‐axis). Error bars represent 95% confidence intervals. The solid line indicates a 1:1 correlation between mean estimates, while dotted lines represent the actual estimated relationship between the mean density of the two models. Estimates are shown from early spring 2020 (February 01, 2020 to March 26, 2020) and winter 2021–2022 (December 01, 2021 to February 01, 2022), as we were not able to conduct random snow tracking surveys in winter 2020–2021 due to COVID‐19. The two colors represent the two approaches to data collection: snow track surveys and camera traps. Each species is represented by a different shape at the point estimate. Please note the pseudo‐log scale of the *y*‐axis.

We developed a total of 56 density estimates across four species, 3 years, and 5 methods (Appendix D). Conventional snow track surveys estimated higher densities than the random surveys, as indicated in the positive intercept (Table 1). All camera‐based methods had little bias relative to random snow track surveys, and estimates between camera‐based models and the FMP matched especially well in 2020 for all species except wild boar (Figure 2). Results were less consistent in 2021–2022: estimates of higher density species tending to be greater than FMP‐based estimates, as indicated by the slopes greater than one. All models detected a dramatic decline in wild boar density between spring 2020 and winter 2020–2021. Randomized track surveys produced similar precision to conventional surveys ($\bar{x}$ = 0.48 RSE, *SD* = 0.16 vs. $\bar{x}$ = 0.47 RSE, *SD* = 0.16). The TTE was the most precise ($\bar{x}$ = 0.38 RSE, *SD* = 0.17) across species and years, while the STE was the least precise ($\bar{x}$ = 0.91 RSE, *SD* = 0.61). The precision of REM fell in the middle, averaging 0.51 RSE (*SD* = 0.28) across species and years.

**TABLE 1:** Intercept and slope estimates when regressing conventional snow track and camera‐based estimates of prey density against FMP estimates from randomized surveys conducted over 3 years in the Sikhote‐Alin Zapovednik, Russian Far East. An intercept (*β*_0_) of 0 indicates no relative bias, while a slope of 1 indicates consistent agreement across the range of prey densities.

Model	Parameter	Est.	SE	*p*
FMP conv	*β*_0_	0.38	0.75	0.63
	*slope*	1.23	0.95	0.24
STE	*β*_0_	−0.09	0.85	0.92
	*slope*	1.41	1.08	0.24
REM	*β*_0_	0.01	0.7	0.98
	*slope*	1.96	0.89	0.07
TTE	*β*_0_	0.02	0.58	0.98
	*slope*	2.07	0.74	0.03*

* Statistically significant coefficient for *p* < 0.05.

**FIGURE 3:**
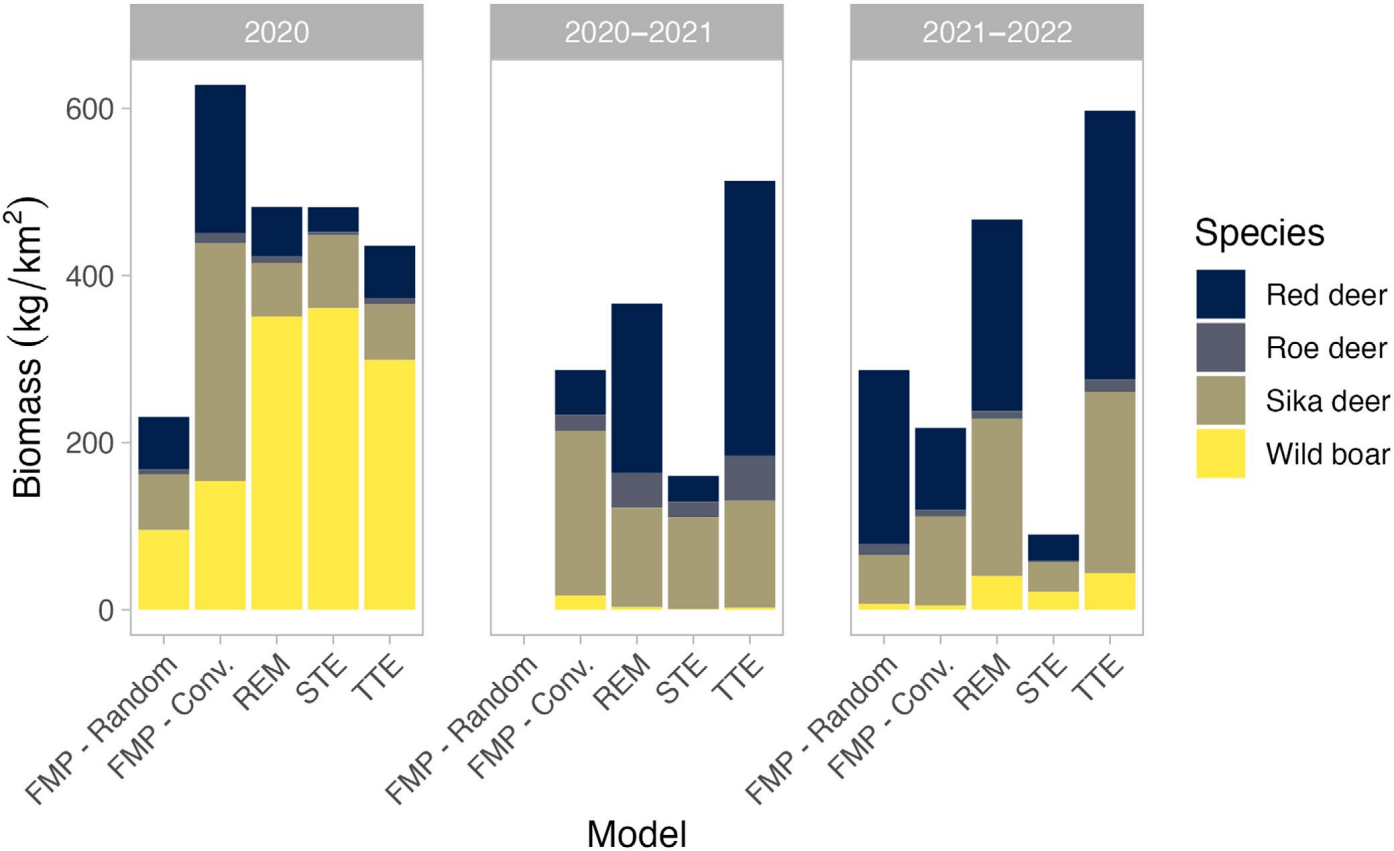
Total prey biomass (kg/km^2^) over 3 years (2020–2022) in southern Sikhote‐Alin Zapovednik, Russian Far East. Total biomass was estimated by multiplying point estimates of species densities by average female weights reported in the literature (Bromley and Kucherenko 1983), then taking their sum. We note that African swine fever arrived in our study area between our first and second years of sampling, causing a large decrease in wild boar density and, therefore, biomass.

Our conversions of point estimates of density to total prey biomass (kg/km^2^) were largely inconsistent across methods in each year (Figure 3). In all three years, estimates of prey biomass using the conventional FMP method indicated that sika deer consistently contributed the most available biomass (45%, 68%, and 49% for the 3 years). However, based on the other methods, wild boar provided the most biomass in spring 2020 while sika deer contributed substantially less. For all methods, roe deer contributed a very small portion of total biomass each year. Wild boar biomass fell dramatically after the arrival of African swine fever (ASF) in summer 2020 (Figure 3), but for many methods (except the STE), the increase in red deer biomass with the western expansion of our study area resulted in a relatively small reduction in total available biomass.

## 4 Discussion

Estimates of population density are an invaluable metric to assess the conservation status of populations and the efficacy of management actions (Williams, Nichols, and Conroy 2002; Nichols and Williams 2006; Karanth et al. 2017). In this study, we found that snow track surveys and camera‐based viewshed density estimators provided roughly similar density estimates of unmarked prey populations in the Russian Far East, indicating that both snow track surveys and camera traps can be used to inform wildlife management and prey‐based tiger conservation. However, we also found that meeting the assumption of all models that camera viewsheds represent the study area was highly demanding of resources and field staff. Even small differences in the estimated densities of large, relatively abundant prey led to stark differences in predicted total biomass. The challenges of meeting strict assumptions and increased differences when converted to biomass lead us to urge caution in the use of these methods to understand available prey biomass and inform tiger conservation in many parts of the species' range. Below, we consider these challenges in more detail.

Both the FMP and viewshed density estimators rely on the assumption that the space sampled by transects/cameras are representative of the study area. In their review of 34 studies comparing REM estimates with reference methods, Palencia et al. (2022) found that studies with targeted camera placement (e.g., on game trails or latrine sites) had positive bias in density estimates due to increased encounter rates, as many species preferentially use roads and trails to travel (e.g., Tanwar, Sadhu, and Jhala 2021; Montalvo et al. 2023). Lyet et al. (2023) similarly found that targeted camera placement can confound the violation of other assumptions: the negative bias in STE density estimates from inflated viewshed measurements was compensated by increased detection rates of cameras on trails for three populations of white‐tailed deer in British Columbia, Canada. In this study, our FMP estimates from surveys along roads and trails largely differed from our representative surveys both in their density estimates and relative species contributions to prey biomass. We therefore strongly recommend representative surveys as an essential part of study design for all methods applied in our study so that survey routes and camera traps accurately sample the heterogeneity in animal distribution across the study area.

All methods also assume that detections are independent. This assumption may be harder to meet for social animals that live in groups. Simulation work by Chauvenet et al. (2017) and Hayashi and Iijima (2022) suggests bias can be introduced by group size; however, the camera detection radii used in these two studies were large (18 and 13 m, respectively), and simulated animals grouped relatively close together; whereas our estimated detection distances were small (ranging 4–7 m), and even for large groups of sika deer or wild boar, it was rare for more than one individual to be within that 4–7 m range. Other studies from the field suggest mixed results: REM estimates of wild boar (a social, grouping species) from Palencia et al. (2022) were generally larger than the reference estimates, but earlier work by Cusack et al. (2015) found that REM estimates of lion density in Serengeti National Park matched well with reference estimates, so long as encounters were considered of individuals, not groups. Lyet et al. (2023) found that STE density estimates became more negatively biased for species with larger group size. Our own results do not offer any clear relationships. Camera‐based estimates of wild boar density in 2020 were higher than snow track estimates, yet estimates of sika deer—a species of larger group size (Stephens, Zaumyslova, Miquelle et al. 2006)—matched well. Differences were more likely due to difficulties in accurately counting the number of individuals in a group based on snow tracks (Stephens, Zaumyslova, Miquelle et al. 2006; Keeping et al. 2018). The effect of group size on estimate accuracy remains unclear and in need of further research.

Two other assumptions related to animal movement rate should be especially considered by managers. First, for the FMP, REM, and TTE, practitioners should assess their ability to obtain independent estimates of movement rate because of this parameter's influence on density estimates (Loonam, Lukacs, et al. 2021; Keeping and Pelletier 2014; Palencia et al. 2022). We believe our use of movement rate estimates from the literature was justified as they came from the same study area; however, these movement rates can vary by season (Stephens, Zaumyslova, Hayward et al. 2006) and year (Waller et al. 2024), whereas we used the same estimates across years. As found by Palencia et al. (2022), we agree the preferred method should be to estimate daily travel distances during the same time period as the survey, whether by snow tracking or using camera‐based methods (Palencia et al. 2019). Second, when applying the STE, practitioners should consider the ability of their camera traps to meet the assumption of instantaneous sampling. For STE estimates to be independent of animal movement rate, the sampling period must be 1 s (Moeller, Lukacs, and Horne 2018; Lyet et al. 2023). Yet if there are gaps between images in the same capture sequence that are greater than 1 s (e.g., due to trigger delays), the estimates are likely to be negatively biased. In our study, the STE estimated consistently lower densities than other estimators during winters 2020–2021 and 2021–2022. One likely explanation was our use of Panthera V7 cameras during these years, which were designed for carnivore monitoring and had gaps in time between images of the same capture sequence. Though developments by Lyet et al. (2023) have increased the applicability of the STE model to motion‐trigger data, we maintain that STE estimates will be most reliable when images are collected with a time‐lapse setting (Moeller, Lukacs, and Horne 2018; Moeller et al. 2023).

For tiger managers seeking to use these methods to understand prey populations, all models are limited by the lack of robust methods to convert prey density estimates to biomass. We found the greatest differences between estimates of total prey biomass when models varied in their density estimates of the most abundant and largest prey. This was most obvious in differences in wild boar biomass in Spring 2020. We recognize that by extrapolating prey biomass from point estimates of density, we ignored the uncertainty associated with each density estimate which would have rendered these differences insignificant. But this has been the convention for over 30 years. Karanth and Sunquist (1992) only presented biomass point estimates due to the imprecision of their estimates of prey density, and others have followed suit (e.g., Miquelle et al. 2010; Zhang, Zhang, and Stott 2013; Upadhyay et al. 2019). There is thus a critical need to develop more robust methods to convert prey density estimates to prey biomass that incorporate uncertainty while still providing precise, unbiased estimates.

All models considered here share the above challenges. But whether tiger managers choose to monitor prey densities with snow track surveys or viewshed density estimators will depend on their resources available, environment, and management context. In Table 2, we summarize the practical advantages and disadvantages of each approach and model. While there are many technical details to consider, we expect the major limitations are likely to be (1) whether there are consistent, suitable snow conditions to conduct track surveys, and (2) whether there are sufficient funds to conduct a camera trap survey and process the large amount of data. Toward this point, we emphasize that the FMP is most useful for researchers and managers with limited resources (e.g., to purchase expensive survey equipment like camera traps) because of the reduced total amount of field work required for the quality of data obtained. In our study, in winter 2021–2022, we traveled 423 km off‐trail to deploy and retrieve cameras, but only walked/skied 162 km off‐trail for representative track surveys. Time to organize, curate, and analyze data was also considerably less. Other studies have found track surveys to be cheaper than other methods like aerial surveys (Keeping et al. 2018). We encourage managers to especially consider the increased labor costs of deploying cameras using a random or systematic study design given the rugged terrain in much of tiger habitat. As managers consider their options, Table 2 can help clarify some of the major advantages and challenges of the models applied here.

**TABLE 2:** Advantages and disadvantages of each model for managers seeking to monitor densities of unmarked wildlife populations, based on our experience in this study. Advantages/disadvantages that apply to all three camera estimators are in cells labeled “All.”

Method	Advantages		Disadvantages	
FMP	Practically no material costs needed to conduct surveys Less effort (off‐trail travel) required for the same level of precision as camera‐based methods Encourages community members with expert tracking skills to participate in wildlife management Time to process data and produce results is much faster than camera‐based methods		Requires appropriate substrate (e.g., snow, sand) conditions to identify species and age of tracks Difficult to plan surveys around fickle weather Need estimate of species movement rate which cannot be estimated from survey itself Relies heavily on accurate and consistent recording of raw data (track encounters) across surveyors	
STE	All: Cameras can be deployed in any environment Raw data (detection events) are easily saved and can be reviewed Familiar technology for tiger managers All parameters estimable from cameras	STE: No estimate of movement rate needed	All: Start‐up costs of purchasing cameras may be prohibitive Considerable effort required to achieve desired level of precision Data processing is time‐consuming Unfamiliar models and assumptions to tiger managers Cannot use “by‐catch” detections of prey during tiger monitoring survey for estimating true densities of prey	STE: Biased low if consistent temporal gaps between photos of same detection event Especially sensitive to viewshed measurement Variance in viewshed yet to be incorporated in model Less precise than REM and TTE
REM		REM: Well‐documented and supported model If independent estimate of movement, only need detection event (not continuous sampling)		REM: Need accurate estimate of species movement rate Not as precise as other movement‐based model (TTE)
TTE		TTE: Consistently produces the most precise estimates If independent estimate of movement, only need detection event (not continuous sampling)		TTE: Need accurate estimate of species movement rate Variance in viewshed yet to be incorporated in model Variance in movement (via period length) yet to be incorporated in model Unclear definition of period length

In conclusion, this study demonstrates that viewshed density estimators offer an alternative to snow track surveys in areas with decreasing and/or inconsistent snowfall. There are challenges with these methods, but they can be accounted for with sufficient resources, proper planning, and survey design. In the context of tiger conservation, we strongly caution not to use “by‐catch” data from cameras placed to monitor tigers to infer prey biomass, as these cameras are nearly always placed on roads or trails to maximize detections of tigers, thus violating the critical assumption of viewshed density estimators that the collective camera viewsheds representatively sample the landscape. Lastly, we encourage future research to develop more robust methods to estimate prey biomass that account for uncertainty in estimates of prey density.

## Author Contributions

**Scott J. Waller:** conceptualization (equal), formal analysis (lead), funding acquisition (supporting), investigation (lead), methodology (lead), writing – original draft (lead), writing – review and editing (equal). **Mark Hebblewhite:** conceptualization (equal), formal analysis (supporting), funding acquisition (supporting), methodology (supporting), writing – review and editing (equal). **Jedediah F. Brodie:** conceptualization (equal), formal analysis (supporting), funding acquisition (supporting), methodology (supporting), writing – review and editing (equal). **Svetlana V. Soutyrina:** conceptualization (supporting), investigation (supporting), methodology (supporting), writing – review and editing (supporting). **Dale G. Miquelle:** conceptualization (equal), funding acquisition (lead), investigation (supporting), methodology (supporting), writing – review and editing (equal).

## Conflicts of Interest

The authors declare no conflicts of interest.

## Supporting information


Data S1.

## Data Availability

Our data come from a Russian federal reserve, and we cannot make it publicly available. Data may be made available upon request to the corresponding author. We also have provided example R code for the FMP, REM, and TTE models in the Supporting Information. For the STE, please refer to the vignette provided by Lyet et al. (2023).

## Appendix A Camera Model Specifications

Table A1 included in “Tables” section below.

**TABLE A1:** Summary of survey efforts during winters 2019–2020 and 2020–2021 for both camera trap surveys and snow track surveys. See “Methods” section for more details about study design. Numbers in parentheses after the camera type indicates the number of models of that brand that were used.

	Spring 2020	Winter 2020–2021	Winter 2021–2022
Study dates	February 01, 2020 to March 26, 2020	December 10, 2020 to February 10, 2021	December 01, 2021 to February 01, 2022
Cams deployed	36	38	45
Cams used	31	36	41
Trap nights	1375	2223	2583
Cam brand	Bushnell (2)	Panthera V7 (1)	Panthera V7 (1)
	Reconyx (2)	Browning Recon Force (1)	Browning Recon Force (1)
	SPromise (2)		
	Browning (2)		

## Appendix B Method to Estimate Effective Detection Distance

We applied a combination of automated (Haucke et al. 2021) and manual distance sampling techniques to estimate the distances to detected animals during winter 2021‐2022. In order to use the DistanceEstimation software, we needed to capture calibration photos during our deployment. After securing each camera in place at each site, we laid out a measuring tape directly in front of the camera. Then, we triggered images at 2, 4, 6, 8, and 10 m, using our fingers to indicate the distance. These images were then used at the “calibration_frames” in the automated distance estimation workflow described in Haucke et al. (2021). (https://github.com/timmh/distance‐estimation/releases). Following Haucke et al.'s suggestion, we used the GIMP software to generate black‐and‐white “calibration_masks.” This gave us the requisite material to run the software, which at the end generates a .csv file with each image name and the estimated distance to the animal.

Next, we verified these distances estimated by the DistanceEstimation software using conventional human distance estimation techniques. We used the same calibration images as a reference for distance. If the distance estimated by the software looked appropriate, we retained the software‐based estimate for that image. Otherwise, we manually estimated the distance ourselves and used the new, manual estimate instead of the software estimate.

Once we finished processing our distance data, we followed the capture probability estimation workflow provided in a vignette by Howe et al. for each species (https://examples.distancesampling.org/Distance‐cameratraps/camera‐distill.html). To estimate effective detection distance (*EDD*), we followed Hofmeester et al. (2017) and took the square root of the product of the capture probability (*p*) and the square of the maximum distance (*w*) used in the estimation of *p* (i.e., after the right‐truncation):

$$\mathrm{EDD}=\sqrt{p\times w^{2}}$$

We were only able to apply this workflow during our third year (winter 2021–2022). During the previous 2 years, we did not yet appreciate the importance of accounting for imperfect detection and were instead using techniques to measure the viewable area (see Moeller et al. 2023 for a more thorough discussion of the components of a camera's viewshed area). As such, we used the estimates of effective detection distance from winter 2021 to –2022 for the calculation of effective detection area of spring 2020 and winter 2020–2021 as well.

## Appendix D Density Estimates by Year, Species, and Model

**Table:**

Species	Year	Model	Density	SE	RSE
Wild boar	2020	FMP random	1.11	0.3	0.3
		FMP trail	1.79	0.79	0.44
		STE	4.2	1.54	0.37
		REM	4.08	1.23	0.3
		TTE	3.48	0.65	0.19
	2020–2021	FMP trail	0.2	0.08	0.41
		STE	0.01	0.03	2.3
		REM	0.04	0.05	1.19
		TTE	0.03	0.03	0.78
	2021–2022	FMP random	0.08	0.06	0.77
		FMP trail	0.06	0.05	0.79
		STE	0.25	0.16	0.64
		REM	0.47	0.37	0.78
		TTE	0.51	0.19	0.38
Red deer	2020	FMP random	0.42	0.23	0.54
		FMP trail	1.19	0.32	0.27
		STE	0.2	0.22	1.07
		REM	0.4	0.2	0.5
		TTE	0.42	0.16	0.37
	2020–2021	FMP trail	0.36	0.15	0.43
		STE	0.21	0.08	0.36
		REM	1.36	0.39	0.29
		TTE	2.21	0.46	0.21
	2021–2022	FMP random	1.4	0.4	0.28
		FMP trail	0.66	0.26	0.39
		STE	0.21	0.07	0.36
		REM	1.54	0.57	0.34
		TTE	2.16	0.8	0.37
Roe deer	2020	FMP random	0.17	0.1	0.6
		FMP trail	0.34	0.17	0.52
		STE	0.1	0.15	1.52
		REM	0.21	0.2	0.95
		TTE	0.2	0.07	0.34
	2020–2021	FMP trail	0.55	0.21	0.38
		STE	0.53	0.32	0.59
		REM	1.2	0.39	0.33
		TTE	1.53	0.33	0.22
	2021–2022	FMP trail	0.22	0.11	0.5
		STE	0.05	0.08	1.61
		REM	0.26	0.15	0.59
		TTE	0.42	0.24	0.56
Sika deer	2020	FMP random	0.9	0.44	0.49
		FMP trail	3.85	1.52	0.39
		STE	1.18	1.06	0.91
		REM	0.87	0.63	0.73
		TTE	0.9	0.43	0.48
	2020–2021	FMP trail	2.66	1.07	0.4
		STE	1.48	1	0.68
		REM	1.6	0.79	0.49
		TTE	1.73	0.47	0.27
	2021–2022	FMP random	0.79	0.38	0.49
		FMP trail	1.44	0.97	0.67
		STE	0.48	0.29	0.6
		REM	2.54	1.25	0.49
		TTE	2.93	1.21	0.41

## References

[ece370747-bib-0001] Ahlswede, S. , E. C. Fabiano , D. Keeping , and K. Birkhofer . 2019. “Using the Formozov–Malyshev–Pereleshin Formula to Convert Mammal Spoor Counts Into Density Estimates for Long‐Term Community‐Level Monitoring.” African Journal of Ecology 57: 177–189.

[ece370747-bib-0002] Brodie, J. F. , A. J. Giordano , E. F. Zipkin , H. Bernard , J. Mohd‐Azlan , and L. Ambu . 2015. “Correlation and Persistence of Hunting and Logging Impacts on Tropical Rainforest Mammals.” Conservation Biology 29: 110–121. 25196079 10.1111/cobi.12389

[ece370747-bib-0080] Bromley, G. F. , and S. P. Kucherenko . 1983. Ungulates of the Southern Far East USSR. Moscow, Russia: Nauka Press.

[ece370747-bib-0003] Cappelle, N. , E. J. Howe , C. Boesch , and H. S. Kühl . 2021. “Estimating Animal Abundance and Effort–Precision Relationship With Camera Trap Distance Sampling.” Ecosphere 12: e03299.

[ece370747-bib-0004] Carpio, A. J. , M. Apollonio , and P. Acevedo . 2021. “Wild Ungulate Overabundance in Europe: Contexts, Causes, Monitoring, and Management Recommendations.” Mammal Review 51: 95–108.

[ece370747-bib-0005] Chauvenet, A. L. M. , R. M. A. Gill , G. C. Smith , A. I. Ward , and G. Massei . 2017. “Quantifying the Bias in Density Estimated From Distance Sampling and Camera Trapping of Unmarked Individuals.” Ecological Modelling 350: 79–86.

[ece370747-bib-0006] Cusack, J. J. , A. Swanson , T. Coulson , et al. 2015. “Applying a Random Encounter Model to Estimate Lion Density From Camera Traps in Serengeti National Park, Tanzania.” Journal of Wildlife Management 79: 1014–1021. 26640297 10.1002/jwmg.902 PMC4657488

[ece370747-bib-0007] Dou, H. , H. Yang , J. L. D. Smith , L. Feng , T. Wang , and J. Ge . 2019. “Prey Selection of Amur Tigers in Relation to the Spatiotemporal Overlap With Prey Across the Sino–Russian Border.” Wildlife Biology 2019: 1–11.

[ece370747-bib-0008] Efford, M. G. , D. L. Borchers , and A. E. Byrom . 2009. “Density Estimation by Spatially Explicit Capture‐Recapture: Likelihood‐Based Methods.” In Modeling Demographic Processes in Marked Populations, 255–269. Berlin, Germany: Springer Science & Business Media.

[ece370747-bib-0009] Efron, B. , and R. J. Tibshirani . 1993. An Introduction to the Bootstrap. New York: Chapman & Hall.

[ece370747-bib-0010] Formozov, A. N. 1932. “Formula for Quantitative Censusing of Mammals by Tracks.” Russian Journal of Zoology 11: 66–69.

[ece370747-bib-0011] Garshelis, D. L. , K. V. Noyce , and V. St‐Louis . 2020. “Population Reduction by Hunting Helps Control Human‐Wildlife Conflicts for a Species That Is a Conservation Success Story.” PLoS One 15: e0237274. 32780755 10.1371/journal.pone.0237274 PMC7418986

[ece370747-bib-0012] Gilbert, N. A. , J. D. J. Clare , J. L. Stenglein , and B. Zuckerberg . 2020. Abundance estimation of unmarked animals based on camera‐trap data. Hoboken, NJ: Blackwell Publishing Inc. 10.1111/cobi.13517 32297655

[ece370747-bib-0014] Gray, T. N. E. , R. Rosenbaum , G. Jiang , et al. 2023. “Restoring Asia's Roar: Opportunities for Tiger Recovery Across the Historic Range.” Frontiers in Conservation Science 4: 1124340.

[ece370747-bib-0015] Harihar, A. , B. Pandav , and D. C. Macmillan . 2014. “Identifying Realistic Recovery Targets and Conservation Actions for Tigers in a Human‐Dominated Landscape Using Spatially Explicit Densities of Wild Prey and Their Determinants.” Diversity and Distributions 20: 567–578.

[ece370747-bib-0016] Haucke, T. , H. S. Kühl , J. Hoyer , and V. Steinhage . 2021. “Overcoming the Distance Estimation Bottleneck in Estimating Animal Abundance with Camera Traps.” Ecological Informatics 68: 101536.

[ece370747-bib-0017] Hayashi, K. , and H. Iijima . 2022. “Density Estimation of Non‐independent Unmarked Animals From Camera Traps.” Ecological Modelling 472: 110100.

[ece370747-bib-0018] Hayward, M. W. , W. Jedrzejewski , and B. Jedrzewska . 2012. “Prey Preferences of the Tiger P Anthera Tigris.” Journal of Zoology 286: 221–231.

[ece370747-bib-0020] Hebblewhite, M. , D. G. Miquelle , H. Robinson , et al. 2014. “Including Biotic Interactions With Ungulate Prey and Humans Improves Habitat Conservation Modeling for Endangered Amur Tigers in the Russian Far East.” Biological Conservation 178: 50–64.

[ece370747-bib-0021] Helle, P. , K. Ikonen , and A. Kantola . 2016. “Wildlife Monitoring in Finland: Online Information for Game Administration, Hunters, and the Wider Public.” Canadian Journal of Forest Research 46: 1491–1496.

[ece370747-bib-0022] Heptner, V. G. , A. A. Nasimovich , and A. G. Bannikov . 1988. Mammals of the Soviet Union: Volume 1. Washington, DC: Smithsonian Institution.

[ece370747-bib-0082] Hofmeester, T. R. , J. M. Rowcliffe , and P. A. Jansen . 2017. “A Simple Method for Estimating the Effective Detection Distance of Camera Traps.” Remote Sensing in Ecology and Conservation 3, no. 2: 81–89. 10.1002/rse2.25.

[ece370747-bib-0023] Hofmeester, T. R. , J. P. G. M. Cromsigt , J. Odden , H. Andrén , J. Kindberg , and J. D. C. Linnell . 2019. Framing Pictures: A Conceptual Framework to Identify and Correct for Biases in Detection Probability of Camera Traps Enabling Multi‐Species Comparison. Hoboken, NJ: John Wiley and Sons Ltd. 10.1002/ece3.4878 PMC6392353 30847112

[ece370747-bib-0024] Howe, E. J. , S. T. Buckland , M. L. Després‐Einspenner , and H. S. Kühl . 2017. “Distance Sampling With Camera Traps.” Methods in Ecology and Evolution 8: 1558–1565.

[ece370747-bib-0025] Hutchinson, J. M. C. , and P. M. Waser . 2007. “Use, misuse and extensions of “ideal gas” models of animal encounter.” Biological Reviews 82: 335–359. 17624958 10.1111/j.1469-185X.2007.00014.x

[ece370747-bib-0079] Intergovernmental Panel on Climate Change (IPCC) . 2022. “Climate Change 2022: Impacts, Adaptation, and Vulnerability.” In Contribution of Working Group II to the Sixth Assessment Report of the Intergovernmental Panel on Climate Change, edited by H.‐O. Pörtner . Cambridge: Cambridge University Press.

[ece370747-bib-0026] Karanth, K. U. 1995. “Estimating Tiger *Panthera tigris* Populations From Camera‐Trap Data Using Capture‐Recapture Models.” Biological Conservation 71: 333–338.

[ece370747-bib-0027] Karanth, K. U. , and J. D. Nichols . 2002. Monitoring Tigers and Their Prey: A Manual for Wildlife Researchers, Managers and Conservationists in Tropical Asia. Bangalore, India: Centre for Wildlife Studies.

[ece370747-bib-0028] Karanth, K. U. , N. S. Kumar , and K. K. Karanth . 2020. “Tigers Against the Odds: Applying Macro‐Ecology to Species Recovery in India.” Biological Conservation 252: 108846.

[ece370747-bib-0029] Karanth, K. U. , and M. E. Sunquist . 1992. “Population Structure, Density and Biomass of Large Herbivores in the Tropical Forests of Nagarahole, India.” Page Source: Journal of Tropical Ecology 8: 21–35.

[ece370747-bib-0030] Karanth, U. K. , J. D. Nichols , J. M. Goodrich , et al. 2017. “Role of Monitoring in Global Tiger Conservation.” In Methods for Monitoring Tiger and Prey Populations, edited by K. Karanth and J. Nichols , 1–13. Singapore: Springer.

[ece370747-bib-0031] Keeping, D. , J. H. Burger , A. O. Keitsile , et al. 2018. “Can Trackers Count Free‐Ranging Wildlife as Effectively and Efficiently as Conventional Aerial Survey and Distance Sampling? Implications for Citizen Science in the Kalahari, Botswana.” Biological Conservation 223: 156–169.

[ece370747-bib-0032] Keeping, D. , and R. Pelletier . 2014. “Animal Density and Track Counts: Understanding the Nature of Observations Based on Animal Movements.” PLoS One 9: 1–11. 10.1371/journal.pone.0096598 PMC4037204 24871490

[ece370747-bib-0033] Kerley, L. L. , A. S. Mukhacheva , D. S. Matyukhina , E. Salmanova , G. P. Salkina , and D. G. Miquelle . 2015. “A Comparison of Food Habits and Prey Preference of Amur Tiger ( *Panthera tigris altaica* ) at Three Sites in the Russian Far East.” Integrative Zoology 10: 354–364. 25939758 10.1111/1749-4877.12135

[ece370747-bib-0034] Loonam, K. E. , P. E. Lukacs , D. E. Ausband , M. S. Mitchell , and H. S. Robinson . 2021. “Assessing the Robustness of Time‐To‐Event Abundance Assessing the Robustness of Time‐To‐Event Abundance Estimation.” Ecological Applications 31: e02388. 34156123 10.1002/eap.2388

[ece370747-bib-0035] Loonam, K. E. , D. E. Ausband , P. M. Lukacs , M. S. Mitchell , and H. S. Robinson . 2021. “Estimating Abundance of an Unmarked, Low‐Density Species Using Cameras.” Journal of Wildlife Management 85: 87–96.

[ece370747-bib-0036] Lyet, A. , S. Waller , T. Chambert , et al. 2023. “Estimating Animal Density Using the Space‐To‐Event Model and Bootstrap Resampling With Motion‐Triggered Camera‐Trap Data.” Remote Sensing in Ecology and Conservation 10: 141–155.

[ece370747-bib-0037] Miquelle, D. G. , J. M. Goodrich , E. N. Smirnov , et al. 2010. “Amur Tiger: A Case Study of Living on the Edge.”

[ece370747-bib-0038] Miquelle, D. G. , A. S. Mukhacheva , E. V. Bragina , et al. 2024. “Rehabilitating Tigers for Range Expansion: Lessons From the Russian Far East.” Journal of Wildlife Management: e22691.

[ece370747-bib-0039] Miquelle, D. G. , E. N. Smirnov , H. B. Quigley , M. G. Hornocker , I. G. Nikolaev , and E. N. Matyushkin . 1996. “Food Habits of Amur Tigers in Sikhote‐Alin Zapovednik and the Russian Far East, and Implications for Conservation.” Journal of Wildlife Research 1: 138–147.

[ece370747-bib-0040] Moeller, A. K. , and P. M. Lukacs . 2021. “spaceNtime: An R Package for Estimating Abundance of Unmarked Animals Using Camera‐Trap Photographs.” Mammalian Biology 102: 581–590.

[ece370747-bib-0041] Moeller, A. K. , P. M. Lukacs , and J. S. Horne . 2018. “Three Novel Methods to Estimate Abundance of Unmarked Animals Using Remote Cameras.” Ecosphere 9: e02331.

[ece370747-bib-0042] Moeller, A. K. , S. J. Waller , N. J. DeCesare , M. C. Chitwood , and P. M. Lukacs . 2023. “Best Practices to Account for Capture Probability and Viewable Area In Camera‐Based Abundance Estimation.” Remote Sensing in Ecology and Conservation 9: 152–164.

[ece370747-bib-0043] Montalvo, V. H. , C. Sáenz‐Bolaños , J. C. Cruz‐Díaz , J. M. Kamilar , E. Carrillo , and T. K. Fuller . 2023. “Effects of Camera Trap Placement on Photo Rates of Jaguars, Their Prey, and Competitors in Northwestern Costa Rica.” Wildlife Society Bulletin 47: e1428.

[ece370747-bib-0044] Morelle, K. , J. Bubnicki , M. Churski , J. Gryz , T. Podgórski , and D. P. J. Kuijper . 2020. “Disease‐Induced Mortality Outweighs Hunting in Causing Wild Boar Population Crash After African Swine Fever Outbreak. Frontiers in Veterinary.” Science 7: 378. 10.3389/fvets.2020.00378 PMC7399055 32850993

[ece370747-bib-0045] Morin, D. J. , J. Boulanger , R. Bischof , et al. 2022. “Comparison of Methods for Estimating Density and Population Trends for Low‐Density Asian Bears.” Global Ecology and Conservation 35: 1–21.

[ece370747-bib-0046] Nakashima, Y. , K. Fukasawa , and H. Samejima . 2018. “Estimating Animal Density Without Individual Recognition Using Information Derivable Exclusively From Camera Traps.” Journal of Applied Ecology 55: 735–744.

[ece370747-bib-0047] Nasi, R. , A. Taber , and N. Van Vliet . 2011. “Empty forests, empty stomachs? Bushmeat and livelihoods in the Congo and Amazon Basins.” International Forestry Review 13, no. 3: 355–368.

[ece370747-bib-0048] Nichols, J. D. , and B. K. Williams . 2006. “Monitoring for Conservation.” Trends in Ecology & Evolution 21: 668–673. 16919361 10.1016/j.tree.2006.08.007

[ece370747-bib-0049] Niedballa, J. , R. Sollmann , A. Courtiol , and A. Wilting . 2016. “camtrapR: An R Package for Efficient Camera Trap Data Management.” Methods in Ecology and Evolution 7: 1457–1462.

[ece370747-bib-0050] Palencia, P. , P. Barroso , J. Vicente , T. R. Hofmeester , J. Ferreres , and P. Acevedo . 2022. “Random Encounter Model Is a Reliable Method for Estimating Population Density of Multiple Species Using Camera Traps.” Remote Sensing in Ecology and Conservation 8: 670–682.

[ece370747-bib-0051] Palencia, P. , J. M. Rowcliffe , J. Vicente , and P. Acevedo . 2021. “Assessing the Camera Trap Methodologies Used to Estimate Density of Unmarked Populations.” Journal of Applied Ecology 58: 1583–1592.

[ece370747-bib-0052] Palencia, P. , J. Vicente , P. Barroso , J. Barasona , R. C. Soriguer , and P. Acevedo . 2019. “Estimating Day Range From Camera‐Trap Data: The animals' Behaviour as a Key Parameter.” Journal of Zoology 309: 182–190.

[ece370747-bib-0053] Pascual‐Rico, R. , Z. Morales‐Reyes , N. Aguilera‐Alcalá , et al. 2021. “Usually Hated, Sometimes Loved: A Review of Wild ungulates' Contributions to People.” Science of the Total Environment 801: 149652. 34438159 10.1016/j.scitotenv.2021.149652

[ece370747-bib-0054] Qi, J. , J. Gu , Y. Ning , et al. 2021. “Integrated Assessments Call for Establishing a Sustainable Meta‐Population of Amur Tigers in Northeast Asia.” Biological Conservation 261: 109250.

[ece370747-bib-0081] R Core Team . 2023. R: A Language and Environemnt for Statistical Computing. Vienna, Austria: R Foundation for Statistical Computing. https://www.R‐project.org/.

[ece370747-bib-0055] Razenkova, E. , M. Dubinin , A. M. Pidgeon , et al. 2023. “Abundance Patterns of Mammals Across Russia Explained by Remotely Sensed Vegetation Productivity and Snow Indices.” Journal of Biogeography 50: 932–946.

[ece370747-bib-0057] Ripple, W. J. , T. M. Newsome , C. Wolf , et al. 2015. “Collapse of the World's Largest Herbivores.” Science Advances 1, no. 4: e1400103. 26601172 10.1126/sciadv.1400103 PMC4640652

[ece370747-bib-0058] Robinson, J. G. , and E. L. Bennett . 2004. “Having your wildlife and eating it too: An analysis of hunting sustainability across tropical ecosystems.” Animal Conservation Forum 7: 397–408.

[ece370747-bib-0059] Rowcliffe, J. M. , J. Field , S. T. Turvey , and C. Carbone . 2008. “Estimating Animal Density Using Camera Traps Without the Need for Individual Recognition.” Journal of Applied Ecology 45: 1228–1236.

[ece370747-bib-0060] Rowcliffe, J. M. , R. Kays , B. Kranstauber , C. Carbone , and P. A. Jansen . 2014. “Quantifying Levels of Animal Activity Using Camera Trap Data.” Methods in Ecology and Evolution 5: 1170–1179.

[ece370747-bib-0061] Rowcliffe, M. J. , C. Carbone , P. A. Jansen , R. Kays , and B. Kranstauber . 2011. “Quantifying the Sensitivity of Camera Traps: An Adapted Distance Sampling Approach.” Methods in Ecology and Evolution 2: 464–476.

[ece370747-bib-0062] Royle, J. A. , J. D. Nichols , K. U. Karanth , and A. M. Gopalaswamy . 2009. “A Hierarchical Model for Estimating Density In Camera‐Trap Studies.” Journal of Applied Ecology 46: 118–127.

[ece370747-bib-0063] Saisamorn, A. , S. Duangchantrasiri , M. Sornsa , W. Suksavate , A. Pattanavibool , and P. Duengkae . 2024. “Recovery of Globally Threatened Ungulate Species in Huai Kha Khaeng Wildlife Sanctuary, Thailand.” Global Ecology and Conservation 53: e03012.

[ece370747-bib-0064] Sanderson, E. W. , D. G. Miquelle , K. Fisher , et al. 2023. “Range‐Wide Trends in Tiger Conservation Landscapes, 2001–2020.” Frontiers in Conservation Science 4: 1191280.

[ece370747-bib-0065] Schaller, G. B. 1967. The Deer and the Tiger. Chicago and London: University of Chicago Press.

[ece370747-bib-0066] Simcharoen, A. , T. Savini , G. A. Gale , et al. 2014. “Female Tiger *Panthera tigris* Home Range Size and Prey Abundance: Important Metrics for Management.” Oryx 48: 370–377.

[ece370747-bib-0067] Stephens, P. A. , O. Y. Zaumyslova , G. D. Hayward , and D. G. Miquelle . 2006. “Analysis of the Long‐Term Dynamics of Ungulates in Sikhote‐Alin Zapovednik, Russian Far East.”

[ece370747-bib-0068] Stephens, P. A. , O. Y. Zaumyslova , D. G. Miquelle , A. I. Myslenkov , and G. D. Hayward . 2006. “Estimating Population Density From Indirect Sign: Track Counts and the Formozov‐Malyshev‐Pereleshin Formula.” Animal Conservation 9: 339–348.

[ece370747-bib-0069] Tanwar, K. S. , A. Sadhu , and Y. V. Jhala . 2021. “Camera Trap Placement for Evaluating Species Richness, Abundance, and Activity.” Scientific Reports 11: 1–11. 34845287 10.1038/s41598-021-02459-w PMC8630032

[ece370747-bib-0070] Tiger Conservation Coalition . 2021. “Securing a viable future for the tiger.”

[ece370747-bib-0071] Upadhyay, H. S. , S. Behera , S. K. Dutta , H. K. Sahu , and J. Sethy . 2019. “A Viable Tiger Population in Similipal Tiger Reserve, India? Calculating if the Ungulate Prey Base Is Limiting.” Wildlife Biology 1: 1–7.

[ece370747-bib-0072] Waller, S. J. , K. Morelle , I. V. Seryodkin , et al. 2024. “Resource‐Driven Changes in Wild Boar Movement and Their Consequences for the Spread of African Swine Fever in the Russian Far East.” Wildlife Biology: e01276.

[ece370747-bib-0073] Walston, J. , J. G. Robinson , E. L. Bennett , et al. 2010. “Bringing the Tiger Back From the Brink‐The 6 % Solution.” PLoS Biology 8: e1000485. 20856904 10.1371/journal.pbio.1000485 PMC2939024

[ece370747-bib-0075] Wilkie, D. S. , M. Wieland , and J. R. Poulsen . 2019. “Unsustainable vs. Sustainable Hunting for Food in Gabon: Modeling Short‐and Long‐Term Gains and Losses.” Frontiers in Ecology and Evolution 7: 357.

[ece370747-bib-0076] Williams, B. K. , J. D. Nichols , and M. J. Conroy . 2002. Analysis and Management of Wildlife Populations. New York, NY: Academic Press.

[ece370747-bib-0077] WWF, Panthera, WCS, and UNDP . 2018. “Jaguar 2030 Roadmap.”

[ece370747-bib-0078] Zhang, C. , M. Zhang , and P. Stott . 2013. “Does Prey Density Limit Amur Tiger *Panthera tigris altaica* Recovery in Northeastern China?” Wildlife Biology 19: 452–461.

